# Recovery from a Bad Bayonet Wound

**Published:** 1855-02

**Authors:** 


*Recovery from a bad Bayonet Wound.*—A gunner and driver of Royal Artillery had made an attack upon his sargeant, and inflicted two superficial wounds upon one leg and arm. The culprit then rushed through the barrack-square to escape his pursuers, when the sentry on duty at the gate interposed himself with the carbine, in the attitude of "charge bayonets," to obstruct him. The man, as he was rushing through the narrow passage with an impetus which he could not control in time, threw himself (not premeditated it may be observed) with great force upon the bayonet of the sentry, which entered his body an inch to the left of the ensiform cartilage, and passing through the abdomen, emerged by its point to the left of and close to the spinal column some inches lower down. He was seen by Mr. Gallway two minutes after, and was found sitting quite unconcernedly on a form in the guard room. The openings of exit and entrance are apparent, and the bayonet itself was found bent by the violence to which it had been exposed. The man marched in a quarter of an hour afterward, to the hospital, three-quarters of a mile, and at the end of a fortnight was discharged from the same to be placed upon trial for his life. The day after his admission, his urine was a little bloody, and subsequently there was a general anæsthesia of the walls of the thorax and abdomen, which lasted, however, but for awhile. With these exceptions, the injury was not followed by a symptom: nor did the subject of it require a dose of medicine for his recovery. Mr. Gallway very justly attributes much of the happy result in this case to the accident having happened before dinner.—*Association Journal.*

